# Eating Habits, Risk of Breast Cancer, and Diet-Dependent Quality of Life in Postmenopausal Women after Mastectomy

**DOI:** 10.3390/jcm11154287

**Published:** 2022-07-23

**Authors:** Małgorzata Socha, Krzysztof A. Sobiech

**Affiliations:** Department of Human Biology, Wroclaw University of Health and Sport Sciences, al. I. J. Paderewskiego 35, 51-612 Wroclaw, Poland; krzysztof.sobiech@awf.wroc.pl

**Keywords:** breast cancer, risk factors, nutrition, cancer prevention, lifestyle, quality of life

## Abstract

The present study examined dietary risk factors for breast cancer, their association with quality of life, and changes in eating habits in postmenopausal women after mastectomy. The study included 210 women with histologically confirmed invasive breast cancer and 225 women without a cancer diagnosis. Questionnaire data on frequency of intake of 40 different foods, the Block Food Frequency Questionnaire, and SF-36 for evaluation of quality of life were used. All questionnaire data in the patient group were collected after diagnosis. Questions about eating habits covered two time points—before breast cancer diagnosis and after completion of treatment. Logistic regression was applied to calculate the odds ratios of breast cancer risk and 95% confidence intervals. A significant positive association was found between the risk of breast cancer and more frequent intake of red meat, smoked products, offal, animal fat, white bread, potatoes, and sweets, high intake of total fat, and low consumption of dietary fibre. Foods that were inversely associated with the risk of breast cancer included fish, vegetables, fruit, wholemeal bread, and groats. The relationship between quality of life and dietary habits according to the Block Eating Frequency Questionnaire was analysed using multiple regression. It was shown that high intake of total fat reduces the quality of life in its mental components. We observed a positive change in eating habits after cancer diagnosis, albeit not always to the level in the control group. As an important lifestyle component, the diet is of great significance for primary prevention of breast cancer as well as for improving the quality of life of breast cancer patients.

## 1. Introduction

Data published in 2020 show that breast cancer in women is the most commonly diagnosed cancer worldwide, with an estimated 2.3 million new cases (11.7%) per year. A high breast cancer incidence can be observed in Australia, New Zealand, North America, and Europe [1]. Breast cancer is the most common cancer in women in Poland. In 2016, breast cancer cases comprised 22.8% of all registered malignancies, with a mortality rate of 15.5% [2]. The changes observed in recent years in cancer incidence and mortality in Poland are the result of demographic changes, the impact of risk factors, and the level of participation in screening programs [3].

Established risk factors for breast cancer include sex, age, ethnicity, family history of breast cancer, gene mutations, history of benign breast disease, reproductive history, elevated levels of endogenous sex hormones, and lifestyle factors—alcohol consumption, smoking, poor eating habits, and obesity in postmenopausal women [4,5]. Results from large prospective epidemiological studies (*n* = 61,335; mean follow-up time 11.4 years) have shown that weight loss in postmenopausal women is associated with a lower risk of breast cancer (hazard ratio, 0.88; 95% confidence interval, 0.78–0.98; *p* = 0.020) [6]. It has been proven that a lifestyle involving body mass control, physical activity, and moderate alcohol consumption reduces the risk of breast cancer by about 30% [7]. The main causes of overweight and obesity are poor eating habits and lack of physical activity. These factors are closely interrelated, and research results confirm that dietary patterns can play a significant role in breast cancer prevention [7,8,9]. Generalised meta-analyses concerning nutrition and breast cancer incidence do not provide consistent evidence on the preventive effects of diet [10,11]. Conflicting results of epidemiological studies indicate regional differentiation of breast cancer risk factors, including those related to dietary patterns. Research on eating habits of breast cancer patients from different regions of the world seems to be justified. 

It has been shown that the diet of most adult Poles is not balanced; healthy eating was found only in 15% of them [12]. The diet of Polish women is characterised by a high intake of fats, meat and meat products, fast food, salt, sugar, sweets, and sugar-sweetened soft drinks, as well as low consumption of fish, milk, dairy products, whole-grain cereals, vegetables, and fruit [13]. Fats constitute a mean of 35% of total calories consumed by Poles (reference range of 20–35%), including saturated fatty acids (15%) and polyunsaturated fatty acids (6%), and the dietary fibre intake averages 17 g/day (WHO reference range > 25 g/day) [14]. A report of the Public Opinion Research Center [15] showed that the dietary habits of Poles have not changed in 16 years, and 57% of the respondents said that their eating habits were similar to those of their parents. The results of the study conducted by Suliga et al. [13] suggest that adherence to the “traditional-carbohydrate” dietary pattern by Poles, characterised by a higher intake of refined grains, potatoes, sugar and sweets, is associated with a higher risk of abdominal obesity and triglyceridemia.

Earlier detection and improvements in breast cancer treatment positively affect the cancer survival rate. Studies into quality of life and factors affecting the mental status of breast cancer patients are of great significance [16]. There is strong evidence that an appropriate dietary pattern can not only play a significant role in breast cancer prevention but also improve the quality of life in women after mastectomy [17,18]. Therefore, healthy nutrition and physical activity are recommended during breast cancer treatment to improve the health and well-being. Research shows that a large proportion of women after a breast cancer diagnosis comply with recommendations of health care professionals and change changes their dietary habits [18].

The present study examined dietary risk factors of breast cancer, their association with quality of life, and changes in eating habits in postmenopausal women after mastectomy.

## 2. Materials and Methods

### 2.1. Participants

Participants were recruited from post-mastectomy women’s clubs in south-western Poland. A description of the parent study project and detailed socio-demographic characteristics of all study participants can be found in a previous publication [19]. Data from 210 postmenopausal women aged 61.9 (±7.8) years with histologically confirmed invasive breast cancer who fully completed the nutrition questionnaires were included in the current paper. All questionnaire data in the patient group were collected after diagnosis and completion of treatment. All the women had undergone mastectomy and completed treatment. Their mean age at the time of the surgery was 55.7 (±6.9) years; 173 (82.4%) of women had radical mastectomy and only 37 (17.6%) partial mastectomy. The comparison group was collected at the same time as the patient group, and was matched to cases based on age and region of residence. The control consisted of women without any diagnosed malignancies, who had not recently changed their diet. Women were recruited from family health centres and seniors’ meeting clubs from the same localities as the post-mastectomy women’s clubs. Of the 254 women recruited, 29 did not fully complete the dietary questionnaires. The final control group included 225 postmenopausal women aged 63.2 (±8.2) years.

The women were classified as post-menopausal if they stopped menstruating a minimum of one year before the beginning of the study, or if they were aged ≥ 55 years. 

All participants volunteered to take part in the study and provided their written informed consent in accordance with the Declaration of Helsinki. The study was approved by the Research Bioethics Committee of the University School of Physical Education in Wrocław, Poland (Reference No. 27/2014).

### 2.2. Procedures

The basic anthropometric measurements included body height, body weight, and BMI (body mass (kg)/body height (m^2^)). The participants completed a survey questionnaire on socio-demographic characteristics (age, place of residence, education, marital status, occupation); reproductive factors (age at menarche, age at menopause, number of full-term pregnancies, breast feeding); as well as family history of breast cancer and the use of hormone replacement therapy.

The participants also answered questions on the incidence of long-term psychological stress, regular physical activity, and eating habits in the study group at two time points—several years before breast cancer diagnosis and after completion of treatment. The use of pre-disease dietary history is justified on the grounds that dietary changes may have occurred after diagnosis that do not reflect the typical diet consumed prior to breast cancer [20]. Distant dietary habits may be more important than recent food intake in predicting diet-related cancer risk [21]. Women in the control group were asked about their eating habits over the past few years up to the time of the interview.

The participants provided data on their weekly intake of 40 food products: red meat, cured meat, poultry, smoked products, fried products, offal, animal fat, white bread, wholemeal bread, potatoes, sugar, cake or desserts, sweets, fish, raw vegetables, boiled vegetables, fruit, groats, skimmed milk, whole milk, low-fat cottage cheese, cottage cheese, sweetened yogurt, natural yogurt or kefir, hard or processed cheese, legumes, vegetable oil, margarine, butter, eggs, pasta or white rice, salt, salty snacks, spring water, freshly squeezed juice, extracted juice, sweet soft drinks, strong tea, strong coffee, alcohol.

Women reported their usual frequency of food (portion, cup) and alcohol consumption (having at least one drink) as the number of times per week. One drink was defined as a small beer (330 mL) or a glass of wine (125 mL) or a glass of vodka, liquor, or other spirits (30 mL). In order to determine the frequency of total fat and dietary fibre intake, they completed the Block Food Frequency Questionnaire [22] with the following reference ranges: under 22 points—healthy fatty diet; 22–24 points—light fatty diet; 25–27 points—fatty diet; and over 27 points—very fatty diet. More than 30 points indicated intake of a sufficient amount of fruit and vegetables, i.e., a diet rich in dietary fibre. The range of 20–29 points signified a recommended increase of intake of vegetables, fruit, and whole grain products. Low intake of fruit, vegetables, and other foodstuffs rich in dietary fibre was indicated by a sum of points below 20.

Quality of life was assessed using a standardised 36-item Short Form Health Survey questionnaire. Permission was obtained from QualityMetric Incorporated to use the Polish version of the questionnaire (IQOLA SF-36v2 Standard, Poland). SF-36 is commonly applied for subjective assessment of the health status of both healthy patients and patients with breast cancer [23]. It consists of 11 questions including 36 items that allow assessment of quality of life in four concepts of physical health status: Physical Functioning (PF), Role Physical (RP), Bodily Pain (BP), and General Health (GH); and four concepts of mental health status: Vitality (VT), Social Functioning (SF), Role Emotional (RE), and Mental Health (MH). Each concept can be scored from 1 to 100, with the lowest score signifying the lowest quality of life. The questionnaire permits a full assessment of the Physical Component Summary (PCS) and Mental Component Summary (MCS).

### 2.3. Statistical Analysis

All statistical calculations were performed using the Statistica software package (version 13.3, license from StatSoft Polska, Kraków, Poland). The differences between two samples of independent variables (cases vs. controls) were checked with Student’s *t*-test for continuous variables and the chi-squared test for qualitative variables. The odds ratio (OR) for breast cancer risk and the 95% confidence interval (CI) were calculated using logistic regression analysis. The dichotomous variable was breast cancer incidence. The independent variables were the consumed food products (frequency/week) and the sum of points for fat and dietary fibre consumption. The independent variables were categorised based on frequency/week. For assessing the statistical significance of predictors in the logistic regression model for breast cancer risk, two-sided chi-square Wald tests were used. Tests for trend were performed by inputting the dietary variables as continuous variables. The relationships between dietary components and breast cancer risk were examined after adjusting for age and various potential confounding variables such as, family history of breast cancer, place of residence, education level, occupation, long-term stress, age at menarche, age at first birth, parity, breastfeeding duration, age at menopause, hormone replacement therapy, current BMI, alcohol intake, and physical activity. To compare changes in eating habits (frequency per week) between groups (cases before diagnosis vs. cases after diagnosis and treatment vs. control group) a single-factor ANOVA was used. When the ANOVA result was significant, the significance of differences between the groups was checked with Tukey’s test. The relationship between the quality of life of post-mastectomy women who completed treatment and the frequency of fat and dietary fibre intake was analysed using multiple regression. The following variables were included in the regression analysis: age, place of residence, education, occupation, marital status, long-term stress, parity, current body mass index, alcohol intake, physical activity, and foods. The level of statistical significance was set at *p* < 0.05.

## 3. Results

Table 1 presents the general characteristics of women in the study sample. There were differences between the cases and controls in their age at menopause, body mass, BMI, duration of breast feeding, occupation, family history of breast cancer and long-term stress. No statistically significant differences were found between cases and controls in body height, age at menarche, place of residence, marital status, full-term pregnancies, breast feeding, the use of hormone replacement therapy, or regular physical activity.

Table 2 presents the results of the logistic regression analysis. Fourteen out of forty examined foods (all in Table 3) were significantly correlated with the risk of breast cancer incidence. Frequent intake of red meat, smoked products, offal, animal fat (lard, bacon), white bread, potatoes, sugar, cakes, and desserts increased the risk. On the other hand, a high frequency of consumption of fish, raw and boiled vegetables (excluding potatoes), fruit, wholemeal bread, and groats reduced the risk significantly. The other food products from Table 3 showed no statistically significant association with the risk of breast cancer. The risk was significantly higher in women who followed a high-fat diet poor in dietary fibre (Table 2). In both cases and controls, there were no women who followed a diet rich in fibre (>30 points) according to the Block Food Frequency Questionnaire.

Table 3 shows differences in frequency of intake of food products between cases and controls and changes in eating habits after breast cancer treatment.

After diagnosis and treatment of neoplastic disease, patients reduced the consumption of food products that increased the risk of breast cancer (see Table 2), including margarine, sweets, fried products, strong tea, and sweetened soft drinks. At the same time, the intake of red meat and potatoes was still significantly higher in cases than in controls. As for foods reducing the risk of breast cancer after diagnosis, there was an increase in consumption of fish, boiled vegetables, wholegrain bread, skimmed milk, natural yogurt, kefir, freshly squeezed juices, and spring water. The intake of groats, vegetable oils, margarine, fried products, and strong tea after breast cancer diagnosis was significantly lower in the group of cases than in the control group. The frequency of intake of poultry, fruit, and raw vegetables before the cancer diagnosis was significantly lower in the study group than in the control group, and it did not change after treatment. However, the total amount of dietary fibre in the diet increased after the breast cancer diagnosis and treatment, but it was still lower than in the control group. In addition, the patients reduced salt and salty snack intake.

The results of multiple regression analysis between quality of life components, according to the SF-36 questionnaire, and the intake of fat and dietary fibre are presented in Table 4. They show that the increased frequency of total fat intake in women after mastectomy lowers the quality of life components of VT, SF, MH, and the Mental Component Summary. There was no correlation between quality of life and the amount of dietary fibre intake in the examined groups of women.

## 4. Discussion

The present study examined dietary risk factors for breast cancer, their association with quality of life, and changes in eating habits in postmenopausal women after mastectomy. We found statistically significant differences between cases and controls in terms of the frequency of consumption of fourteen foods associated with breast cancer risk.

Excessive consumption of animal fat, red meat, sugar, and white flour products, and insufficient amounts of dietary fibre are characteristic for Polish populations [13,15]. The results of the present study confirm the adverse impact of a diet rich in saturated fatty acids on the risk of breast cancer. The percentage of women who consumed red meat and offal three or more times a week, and animal fat (lard, bacon) two or more times a week, was significantly higher in the group of cases than in the group of controls (23.8% vs. 8.4%, 14.3% vs. 4.9%, and 25.2% vs. 8.9%, respectively). A very fatty diet (>27 points in the Block Questionnaire) was followed by as many as 50.5% of women in the study group and 38.2% in the control group. However, the evidence of a relationship between fat intake and breast cancer risk is conflicting [24]. Most likely, it is the type of consumed fat, not its total intake, that is significant. Some studies have indicated a weak positive correlation between the intake of unsaturated fats and the risk of breast cancer. This correlation was more visible in those post-menopausal women who had never used a hormone replacement therapy [25]. In our study, 81% of the patients were not using hormone replacement therapy, which may confirm the above information. Whereas, a British study revealed that a high intake of meat was related to the risk of breast cancer in both pre- and postmenopausal women [26].

A high percentage of women after mastectomy in the present study reported intake of smoked products more than three times a week (18.1% and 5.3% in the control group), which was significantly correlated with the breast cancer risk (OR = 4.14, 95% CI: 1.30–13.18). Tao et al. [27] also showed that a high daily intake of smoked meat (≥4.44 g/d) was significantly correlated with the breast cancer risk, especially in women after menopause (OR = 3.13, 95% CI: 1.89–5.17). Smoked products are sources of polycyclic aromatic hydrocarbons, whose activity has been linked to the occurrence of breast cancer [28].

The main components of Polish cuisine are white wheat flour and potatoes, widely present in the Poles’ daily diet. These products feature a high glycemic index. Their consumption as well as sucrose intake leads to rapid increases in blood glucose and release of insulin-like growth factor (IGF), and promotes body mass gains. Epidemiological studies indicate that a reduction of carbohydrates in the diet can effectively reduce the risk of breast cancer [29]. In a Korean study, a correlation between breast cancer incidence and a diet rich in carbohydrates, glycemic index, or glycemic load was not found; however, a protective effect of high intake of brown rice has been shown, especially in overweight postmenopausal women [30]. The case group subjects in the present study reported frequent intake of white bread and potatoes. The odds ratio for these foods with an intake frequency of more than five times a week was OR = 4.26, 95% CI: 2.49–7.28, and OR = 6.86, 95% CI: 2.65–17.80, respectively. On the other hand, the odds ratio for sugar and cakes and desserts with an intake frequency three or more than three times a week was respectively 2.14 (95% CI: 1.24–3.72) and 4.80 (95% CI: 1.61–14.33). Whereas, frequent consumption of wholemeal bread and groats reduced breast cancer risk in the present study, OR = 0.42, 95% CI: 0.17–1.03, and OR = 0.27, 95% CI: 0.11–0.69.

The positive effects of frequent intake of fruit and vegetables on breast cancer incidence are often questioned [31]. A study of a Greek population proved that fruit and vegetables intake was inversely correlated with breast cancer incidence; however, the observed correlation was weaker than expected [32]. However, a study on Chinese women showed that the consumption of fruit was significantly correlated with a lower risk of breast cancer, but a higher intake of vegetables also had a protective effect [24]. The present study revealed the protective role of frequent intake of fruit and vegetables. The odds ratio, with the intake frequency of more than five times a week, for raw and boiled vegetables, excluding potatoes, was OR = 0.34, 95% CI: 0.21–0.56, and OR = 0.47, 95% CI: 0.28–0.79, respectively, and for fruit consumed three or more times a week OR = 0.23, 95% CI: 0.06–0.86. The women in the present study generally followed a diet poor in dietary fibre. Low dietary fibre intake, i.e., below 20 points according to the Block Questionnaire, increased the risk of breast cancer (OR = 2.57, 95% CI: 1.58–4.18). Some authors emphasise that high consumption of fruit and vegetables can lower the risk of overweight and obesity, which are significant risk factors for breast cancer in postmenopausal women [6]. Park et al. [33] during their 7-year observation, observed a 13% reduction in the risk of breast cancer incidence in postmenopausal women who consumed 26 g of dietary fibre a day, compared with women who ate only 11 g a day.

Another dietary component examined in terms of its possible association with the risk of breast cancer in women is consumption of fish, which contain n-3 polyunsaturated fatty acids (n-3 PUFA). A recent prospective cohort study [34] revealed that the intake of marine n-3 PUFA was associated with a 14% reduction of the risk of breast cancer (OR = 0.86, 95% CI: 0.78–0.94), i.e., 5% per 0.1 g/day (OR = 0.95, 95% CI: 0.90–1.00). In the present study, a reduced risk of breast cancer incidence was noted in women who ate fish three or more times a week (OR = 0.33, 95% CI: 0.12–0.84). There are the results of studies that have found no relationship between fish intake and breast cancer risk, as well as those in which the consumption of fish showed a positive association with the examined risk [35]. This issue requires further research; however, lifestyle changes involving dietary adjustments are still routinely recommended in women during and after breast cancer treatment [31].

According to Templeton et al. [36], about one half of breast cancer survivors were interested in actions improving the prognosis, 69% expressed their willingness to be engaged in physical activity more than once a week, 87% paid particular attention to dietary adjustments involving increased fruit intake and reduced fat consumption, and 46% used complementary and alternative medicine. Similar results were obtained by Mohammadi et al. [18], who found positive changes in reduced intake of fast food (90%) and red meat (70%), and increased consumption of fruit (85%) and vegetables (78%) in a group of breast cancer survivors. Additionally, Chinese breast cancer patients in a study by Lei et al. [37] reported significant and long-term changes in food intake after cancer diagnosis, which was in line with current nutritional recommendations. In the present study the women after mastectomy changed their pre-diagnosis eating habits after the breast cancer diagnosis and treatment. In general, they increased the frequency of intake of products lowering the breast cancer risk, and decreased the intake of products that increased the risk (Table 3). The differences in intake frequencies between the cases and controls were not always statistically significant. For instance, the weekly frequency of intake of fruit and raw vegetables did not change after breast cancer treatment, the frequency of dietary fibre intake was still lower, and the intake of red meat and potatoes was statistically significantly higher than in the control group. Long-developed dietary habits are difficult to change, even in the face of disease and with the knowledge of dietary recommendations of health care professionals. Additionally, Mohammadi et al. [18] found that only about 29% of breast cancer survivors followed healthy eating practices, 34% followed moderate eating practices, and 37% followed poor eating practices as based on nutrition guidelines.

The dietary pattern is an important lifestyle component. In the context of nutrition, the assessment of quality of life also becomes significant. It has been proven that physical exercise and healthy eating lead to a reduction of body fat and an improvement in the quality of life of women [38]. A lifestyle change involving physical exercise and a low-calorie, healthy diet resulted in a reduction in depressive symptoms and normalisation of hypothalamic–pituitary–adrenal axis regulation in women who were completing breast cancer treatment [39]. The present study showed that greater frequency of fat intake in total was significantly associated with the lowering of quality of life, mostly in the components of mental status: vitality, social functioning, and mental health. Similarly, Mohammadi et al. [18] found a significant correlation between healthy eating practices and social role, cognitive, and emotional functioning scales.

### Limitations

There are some limitations of our study. In our research we did not take into consideration detailed clinical parameters of the cancer diagnosis, which may modify the functioning of the risk factors and affect the prognosis for the patients. The results of some long-term observations indicate that the preventive role of higher consumption of fruit and vegetables may be confined to some selected types of breast cancer. Jung et al. [40] found no correlation between total intake of fruit and vegetables and the risk of breast cancer in general. However, the consumption of fruit and vegetables did reduce the risk of ER-negative breast cancer. It has been proven that physical exercise and healthy eating lead to a reduction of body fat and an improvement in the quality of life of women with triple-negative breast cancer [41]. Another limitation of this study is related to the period of questionnaire data collection. Interviews with patients were conducted after the diagnosis of breast cancer and the completion of treatment. Controls were matched to cases based on age, place of residence, and answered the survey questions at the same time as the cases. Therefore, we cannot exclude the recall bias in the control group in answering questions about eating habits, the presence of long-term psychological stress, and participation in physical activity from the period before breast cancer diagnosis. A limitation of the study is also the lack of quantitative data concerning the energy value of the diet, easily ingested carbohydrates, and fat intake. 

## 5. Conclusions

As an important lifestyle component, diet is of great significance for primary prevention of breast cancer as well as for improving the quality of life of breast cancer patients. Promotion of changes in eating habits should be the foundation of all cancer prevention programs. 

## Figures and Tables

**Table 1:** Selected characteristics of breast cancer postmenopausal women and the comparison group.

Variable	Cases (*n* = 210)	Controls (*n* = 225)	*p*-Value
Age (years)	61.9 ± 7.8	63.2 ± 8.2	0.070
Age at menarche	13.6 ± 1.6	13.9 ± 1.5	0.094
Age at menopause	48.5 ± 4.6	50.3 ± 4.1	<0.001 *
Body height (cm)	160.9 ± 5.9	160.4 ± 6.5	0.434
Body mass (kg)	73.6 ± 12.2	70.5 ± 11.9	0.008 *
BMI (kg/m^2^)	28.4 ± 4.5	27.4 ± 4.4	0.015 *
Duration of breast feeding (months)	10.8 ± 8.4	13.9 ± 12.7	0.009 *
Place of residence			
village	17 (8.1)	24 (10.7)	
town ≤ 100 000 inhabitants	120 (57.1)	128 (46.2)	
city > 100 000 inhabitants	73 (34.8)	97 (43.1)	0.076
Marital status			
unmarried	7 (3.3)	16 (7.1)	
married	137 (65.2)	124 (55.1)	
widow	52 (24.8)	65 (28.9)	
divorced	14 (6.7)	20 (8.9)	0.168
Occupation			
non-working	37 (17.6)	8 (3.5)	
manual worker	48 (22.9)	51 (22.7)	
white collar worker	121 (57.6)	160 (71.1)	
rural worker	4 (1.9)	6 (2.7)	<0.001 *
Family history of breast cancer			
No	160 (76.2)	209 (92.9)	
Yes	50 (23.8)	16 (7.1)	0.023 *
Pregnancy			
No	18 (8.6)	21 (9.3)	
Yes	192 (91.4)	204 (90.7)	0.736
Breast feeding			
No	48 (22.9)	49 (21.8)	
Yes	162 (77.1)	176 (78.2)	0.879
Hormone replacement therapy			
No	170 (81.0)	172 (76.4)	
Yes	40 (19.0)	53 (23.6)	0.255
Regular physical activity ^a^			
No	153 (72.9)	157 (69.8)	
Yes	57 (27.1)	68 (30.2)	0.520
Long term stress ^a^			
No	93 (44.3)	144 (64.0)	
Yes	117 (55.7)	81 (36.0)	<0.001 *

BMI—body mass index; values are mean ± SD—standard deviation or numbers (%); * *p*-value < 0.05 from chi-square test for categorical variables and Student’s *t*-test for continuous variables; **^a^** in women after mastectomy in the period before diagnosis and treatment of breast cancer.

**Table 2:** Association between dietary components and risk of breast cancer in postmenopausal women.

Product (Frequency per Week)	Cases (*n* = 210)	Controls (*n* = 225)	OR (95% CI)	*p*-Value
	Number (%)			
Red meat				
never	19 (9.1)	44 (19.6)	1.00	
occasionally	67 (31.9)	107 (47.6)	1.55 (0.78–3.07)	0.1519
1–2	74 (35.2)	55 (24.4)	6.22 (2.87–13.47)	<0.001 *
≥3	50 (23.8)	19 (8.4)	7.28 (2.91–18.19)	<0.001 *
*p* for trend			<0.001 *	
Smoked products				
never	21 (10.0)	24 (10.7)	1.00	
occasionally	95 (45.2)	132 (58.7)	0.87 (0.42–1.80)	0.714
1–2	56 (26.7)	57 (25.3)	1.20 (0.54–2.62)	0.684
≥3	38 (18.1)	12 (5.3)	4.14 (1.30–13.18)	0.003 *
*p* for trend			0.001 *	
Offal				
never	45 (21.4)	63 (28.0)	1.00	
occasionally	103 (49.1)	123 (54.7)	1.17 (0.64–2.08)	0.761
1–2	32 (15.2)	28 (12.4)	2.12 (0.93–4.81)	0.406
≥3	30 (14.3)	11 (4.9)	4.57 (1.51–13.87)	0.012 *
*p* for trend			0.001 *	
Animal fat				
never	63 (30.0)	97 (43.1)	1.00	
occasionally	94 (44.8)	108 (48.0)	1.17 (0.70–1.97)	0.273
≥2	53 (25.2)	20 (8.9)	2.68 (1.22–5.87)	0.013 *
*p* for trend			<0.001 *	
Fish				
never	16 (7.6)	7 (3.1)	1.00	
occasionally	99 (47.1)	62 (27.6)	0.68 (0.25–1.83)	0.5117
1–2	85 (40.5)	132 (58.7)	0.27 (0.08–0.76)	0.008 *
≥3	10 (4.8)	24 (10.6)	0.33 (0.12–0.84)	0.021 *
*p* for trend			<0.001 *	
Raw vegetables				
<2	94 (44.8)	62 (27.5)	1.00	
3–5	72 (34.3)	74 (32.9)	0.77 (0.47–1.27)	0.3756
>5	44 (20.9)	89 (39.6)	0.34 (0.21–0.56)	<0.001 *
*p* for trend			0.002 *	
Boiled vegetables				
<2	108 (51.4)	77 (34.2)	1.00	
3–5	59 (28.1)	85 (37.8)	0.53 (0.33–0.85)	0.009 *
>5	43 (20.5)	63 (28.0)	0.47 (0.28–0.79)	0.004 *
*p* for trend			0.001 *	
Fruits				
<3	46 (21.9)	19 (8.4)	1.00	
≥3	164 (78.1)	206 (91.6)	0.23 (0.06–0.86)	0.029 *
*p* for trend			0.005 *	
White bread				
never/occasionally	39 (18.6)	105 (46.7)	1.00	
1–2	29 (13.8)	35 (15.6)	2.07 (1.09–3.93)	0.026 *
3–5	37 (17.6)	28 (12.4)	3.23 (1.68–6.21)	0.001 *
>5	105 (50.0)	57 (25.3)	4.26 (2.49–7.28)	<0.001 *
*p* for trend			<0.001 *	
Wholemeal bread				
never	22 (10.5)	8 (3.6)	1.00	
occasionally	63 (30.0)	28 (12.4)	0.75 (0.29–1.97)	0.557
3–5	85 (40.5)	94 (41.8)	0.42 (0.17–1.03)	0.057 *
≥5	40 (19.0)	95 (42.2)	0.20 (0.08–0.50)	0.001 *
*p* for trend			<0.001 *	
Potatoes				
never/occasionally	7 (3.3)	26 (10.4)	1.00	
1–2	22 (10.5)	54 (24.3)	1.26 (0.44–3.56)	0.845
3–5	89 (42.4)	106 (47.7)	2.68 (1.04–6.57)	0.021 *
>5	92 (43.8)	39 (17.6)	6.86 (2.65–17.80)	0.001 *
*p* for trend			<0.001 *	
Groats				
never	20 (9.5)	10 (4.4)	1.00	
occasionally	89 (42.4)	78 (34.7)	0.45 (0.19–1.05)	0.064
1–2	77 (36.7)	101 (44.9)	0.27 (0.11–0.69)	0.005 *
≥3	24 (11.4)	36 (16.0)	0.23 (0.08–0.67)	0.006 *
*p* for trend			0.005 *	
Sugar				
never/occasionally	50 (23.8)	98 (43.6)	1.00	
1–2	13 (6.2)	17 (7.6)	0.85 (0.41–1.80)	0.666
≥3	147 (70.0)	110 (48.8)	2.14 (1.24–3.72)	0.006 *
*p* for trend			<0.001 *	
Cake/desserts				
never/occasionally	97 (46.2)	130 (57.8)	1.00	
1–2	60 (28.6)	56 (24.9)	2.31 (0.91–5.87)	0.078
≥3	53 (25.2)	39 (17.3)	4.80 (1.61–14.33)	0.004 *
*p* for trend			0.015 *	
Fat (sum of points) ^a^				
<22	34 (16.1)	48 (21.3)	1.00	
22–24	30 (14.3)	45 (20.0)	0.92 (0.45–1.87)	0.053
25–27	40 (19.1)	46 (20.4)	1.16 (0.60–2.25)	0.653
>27	106 (50.5)	86 (38.2)	1.87 (1.08–3.24)	0.025 *
*p* for trend			0.003 *	
Dietary fibre (sum of points) ^a^				
≥20	84 (40.0)	146 (64.9)	1.00	
<20	126 (60.0)	79 (35.1)	2.57 (1.58–4.18)	0.001 *
*p* for trend			0.002 *	

OR—odds ratio; CI—confidence interval; * *p*-value < 0.05 from chi-square test or the chi-square test for trend; ^a^ fat and dietary fibre intake according to the Block Food Frequency Questionnaire [22]. Adjusted for age, family history of breast cancer, place of residence, education, occupation, long-term stress, age at menarche, age at first birth, parity, breast feeding duration, age at menopause, hormone replacement therapy, current body mass index, alcohol intake, and physical activity.

**Table 3:** Frequency of intake of selected food products by women after mastectomy.

Product (Frequency per Week)	Cases (*n* = 210)		Controls (*n* = 225) (C)	*p*-Value ^a^	*p*-Value ^a^	*p*-Value ^a^	ANOVA *p*-Value
	Before Diagnosis (BD)	After Treatment (AT)		BD vs. AT	BD vs. C	AT vs. C	
**Non-healthy dietary choices (based on Table 2)**							
Red meat	1.8 ± 1.9	1.3 ± 1.6	0.9 ± 1.3	0.005 *	<0.001 *	0.009 *	<0.001 *
Smoked products	1.4 ± 1.8	1.0 ± 1.5	0.7 ± 1.3	0.045 *	<0.001 *	0.162	<0.001 *
Offal	1.0 ± 1.7	0.7 ± 1.2	0.5 ± 1.2	0.050	0.001 *	0.391	0.001 *
Animal fat	0.9 ± 1.8	0.5 ± 1.3	0.4 ± 1.2	0.019 *	0.001 *	0.628	0.001 *
White bread	4.5 ± 2.8	3.1 ± 2.9	2.6 ± 2.9	<0.001 *	<0.001 *	0.158	<0.001 *
Potatoes	5.0 ± 2.0	4.2 ± 2.2	3.6 ± 2.0	0.001 *	<0.001 *	0.006 *	<0.001 *
Sugar	4.8 ± 3.0	3.7 ± 3.3	3.3 ± 3.2	0.001 *	<0.001 *	0.557	<0.001 *
Cake/desserts	1.8 ± 2.1	1.2 ± 1.8	1.4 ± 2.0	0.005 *	0.129	0.451	0.007 *
Fat (sum of points) **^b^**	27.5 ± 6.6	24.3 ± 5.8	25.0 ± 5.9	<0.001 *	<0.001 *	0.282	<0.001 *
**Healthy dietary choices (based on Table 2)**							
Fish	1.0 ± 1.2	1.5 ± 1.4	1.7 ± 1.4	0.001 *	<0.001 *	0.359	<0.001 *
Raw vegetables	3.3 ± 2.4	3.5 ± 2.5	4.3 ± 2.5	0.789	<0.001 *	0.002 *	<0.001 *
Boiled vegetables	3.1 ± 2.4	3.6 ± 2.3	4.0 ± 2.3	0.098	0.001 *	0.233	0.001 *
Fruit	5.1 ± 2.4	5.5 ± 2.2	5.9 ± 1.8	0.164	0.001 *	0.168	0.001 *
Wholemeal bread	2.5 ± 2.6	3.9 ± 2.7	4.3 ± 2.6	<0.001 *	<0.001 *	0.226	<0.001 *
Groats	3.5 ± 0.8	1.5 ± 1.6	3.3 ± 0.8	<0.001 *	0.084	<0.001 *	<0.001 *
Dietary fibre (sum of points) ^**b**^	18.6 ± 4.0	19.7 ± 4.4	20.5 ± 3.5	0.015 *	<0.001 *	0.104	<0.001 *
**Other dietary choices included in present study**							
Skimmed milk	1.4 ± 2.4	2.1 ± 2.8	2.1 ± 2.6	0.010 *	0.016 *	0.987	0.004 *
Whole milk	1.6 ± 2.4	1.1 ± 2.0	1.5 ± 2.5	0.151	0.994	0.176	0.106
Low-fat cottage cheese	1.4 ± 1.9	1.6 ± 1.9	1.7 ± 2.1	0.700	0.395	0.869	0.409
Cottage cheese	1.9 ± 2.1	1.5 ± 1.9	1.8 ± 2.1	0.213	0.942	0.355	0.197
Sweetened yogurt	1.3 ± 2.0	1.0 ± 1.7	1.2 ± 1.9	0.222	0.832	0.518	0.238
Natural yogurt/kefir	2.0 ± 2.2	2.5 ± 2.2	2.4 ± 2.2	0.049 *	0.074	0.987	0.048 *
Hard cheese/processed cheese	2.1 ± 2.0	1.9 ± 1.8	2.1 ± 2.1	0.455	0.994	0.512	0.408
Cured meat	4.0 ± 2.3	3.4 ± 2.2	3.6 ± 2.4	0.030 *	0.198	0.670	0.036 *
Poultry	2.6 ± 1.6	2.8 ± 1.4	3.0 ± 1.5	0.412	0.026 *	0.391	0.035 *
Legumes	0.6 ± 1.3	0.6 ± 1.1	0.6 ± 1.1	0.941	0.774	0.936	0.792
Vegetable oil	3.7 ± 2.5	3.8 ± 2.5	4.3 ± 2.4	0.978	0.041 *	0.066	0.026 *
Margarine	1.9 ± 2.6	1.2 ± 2.1	1.9 ± 2.7	0.010 *	0.995	0.006 *	0.003 *
Butter	4.7 ± 2.8	4.3 ± 2.9	4.3 ± 2.9	0.480	0.340	0.972	0.329
Eggs	2.8 ± 1.7	2.5 ± 1.6	2.7 ± 1.8	0.195	0.666	0.643	0.226
Salt	6.1 ± 2.1	5.6 ± 2.5	5.5 ± 2.6	0.146	0.043 *	0.872	0.043 *
Sweets	2.7 ± 2.6	1.9 ± 2.3	2.1 ± 2.4	0.003 *	0.069	0.467	0.003 *
Fried products	2.5 ± 1.8	1.7 ± 1.5	2.0 ± 1.8	<0.001 *	0.012 *	0.116	<0.000 *
Pasta/white rice	2.1 ± 1.6	2.1 ± 1.7	1.9 ± 1.4	0.994	0.497	0.558	0.453
Freshly squeezed juice	1.1 ± 1.9	1.7 ± 2.2	1.3 ± 2.0	0.017 *	0.554	0.182	0.022 *
Extracted juice	0.9 ± 1.9	0.7 ± 1.6	0.8 ± 1.6	0.460	0.914	0.697	0.480
Strong tea	3.4 ± 3.2	2.8 ± 3.1	3.6 ± 3.2	0.092	0.864	0.021 *	0.021 *
Strong coffee	4.3 ± 3.1	3.8 ± 3.3	4.1 ± 3.2	0.186	0.680	0.607	0.216
Sweet soft drinks	1.0 ± 2.0	0.4 ± 1.4	0.3 ± 1.2	<0.001 *	<0.001 *	0.930	<0.001 *
Spring water	4.5 ± 2.9	5.3 ± 2.6	5.5 ± 2.5	0.008 *	<0.001 *	0.628	<0.001 *
Salty snacks	0.2 ± 0.8	0.1 ± 0.6	0.04 ± 0.3	0.330	0.030 *	0.520	0.040 *
Alcohol	0.3 ± 0.9	0.2 ± 0.8	0.2 ± 1.0	0.844	0.926	0.979	0.853

^a^ post hoc Tukey’s test; ^b^ fat and dietary fibre intake according to the Block Food Frequency Questionnaire [22]; * *p*-value < 0.05.

**Table 4:** Results of multiple regression analysis between quality of life components of the short-form health survey (SF-36) questionnaire and fat and dietary fibre intake.

SF-36 (Points)	Independent Variable (Points)	Cases (*n* = 210)			Controls (*n* = 225)		
		β	t	*p*	β	t	*p*
Physical functioning	Fat	−0.39	−1.77	0.078	0.26	1.16	0.249
	Dietary fibre	0.35	0.70	0.486	0.11	0.27	0.784
		β	t	*p*	β	t	*p*
Role-physical	Fat	−0.46	−1.28	0.201	0.15	0.34	0.736
	Dietary fibre	−0.17	−0.29	0.772	−0.61	−0.80	0.426
		β	t	*p*	β	t	*p*
Bodily pain	Fat	−0.26	−1.02	0.309	−0.01	−0.03	0.978
	Dietary fibre	0.34	0.81	0.420	−0.80	−1.56	0.120
		β	t	*p*	β	t	*p*
General health	Fat	−0.32	−1.57	0.117	0.07	0.37	0.713
	Dietary fibre	0.08	0.25	0.800	−0.39	−1.15	0.250
		β	t	*p*	β	t	*p*
Vitality	Fat	−0.49	−2.29	0.023 *	0.12	0.60	0.548
	Dietary fibre	0.06	0.17	0.864	−0.38	−1.05	0.295
		β	t	*p*	β	t	*p*
Social functioning	Fat	−0.68	−2.71	0.007 *	0.19	0.72	0.474
	Dietary fibre	−0.34	−0.83	0.406	0.04	0.08	0.937
		β	t	*p*	β	t	*p*
Role-emotional	Fat	−0.64	−1.70	0.091	0.58	1.54	0.125
	Dietary fibre	0.18	0.30	0.763	0.09	0.13	0.898
		β	t	*p*	β	t	*p*
Mental health	Fat	−0.71	−3.43	0.001 *	0.05	0.29	0.773
	Dietary fibre	0.25	0.74	0.459	0.03	0.08	0.937
		β	t	*p*	β	t	*p*
Physical component summary	Fat	−0.06	−0.71	0.481	0.01	0.14	0.890
	Dietary fibre	0.14	0.95	0.341	−0.21	−1.11	0.270
		β	t	*p*	β	t	*p*
Mental component summary	Fat	−0.37	−3.10	0.002 *	0.09	0.79	0.431
	Dietary fibre	−0.02	−0.09	0.925	0.04	0.20	0.844

β—standardised regression coefficient; * *p*-value < 0.05. Variables included in the analysis: age, place of residence, education, occupation, marital status, long-term stress, parity, current body mass index, alcohol intake, physical activity, and foods listed in Table 3.

## Data Availability

All data generated or analysed during this study are included in this published article, are also available from the corresponding author on reasonable request.
